# Effect of Soybean Isoflavones on Growth Performance, Immune Function, and Viral Protein 5 mRNA Expression in Broiler Chickens Challenged with Infectious Bursal Disease Virus

**DOI:** 10.3390/ani9050247

**Published:** 2019-05-16

**Authors:** Mahmoud Mostafa Azzam, Shou-qun JIANG, Jia-li CHEN, Xia-jing LIN, Zhong-yong GOU, Qiu-li FAN, Yi-bing WANG, Long LI, Zong-yong JIANG

**Affiliations:** 1 Guangdong Key Laboratory of Animal Breeding and Nutrition/Guangdong Public Laboratory of Animal Breeding and Nutrition/The Key Laboratory of Animal Nutrition and Feed Science (South China) of Ministry of Agriculture/State Key Laboratory of Livestock and Poultry Breeding/Institute of Animal Science, Guangdong Academy of Agricultural Sciences, Guangzhou 510640, China; kellychan84@163.com (J.-l.C.); linxiajing728@sohu.com (X.-j.L.); yozhgo917@163.com (Z.-y.G.); fanqiuli_829@163.com (Q.-l.F.); wangyibing001@gmail.com (Y.-b.W.); leeloong1985@sina.com (L.L.); 2 Department of Animal Production College of Food & Agriculture Sciences, King Saud University, Riyadh 11451, Saudi Arabia; mazzam@ksu.edu.sa; 3 Poultry Production Department, Faculty of Agriculture, Mansoura University, Mansoura 35516, Egypt; 4 Agro-biological Gene Research Centre, Guangdong Academy of Agricultural Sciences, Guangzhou 510640, China

**Keywords:** broilers, IBDV, soybean isoflavones, immune function, viral protein 5 mRNA expression

## Abstract

**Simple Summary:**

Infectious bursal disease virus (IBDV) is characterized by inflammation and subsequent atrophy of the bursa of Fabricius and immune suppression. However, nutritional strategies are able to ameliorate the negative effects of viral infections. Therefore, the aim of the present study was to determine the effect of different levels of soybean isoflavones (SI) on broiler chickens challenged with IBDV. Based on the findings, supplemental 10~20 mg/kg SI may have a positive effect on broiler chickens infected with IBDV, probably because SI decrease the severity of bursa lesions and viral protein 5 mRNA expression, and have strong antioxidant activity.

**Abstract:**

A total of 200 one-day-old male broilers were assigned to five groups, and each group consisted of four replicates with 10 birds per replicate. Chicks were fed the basal diet with 0 (non-infected control), 0 (infected control), 10, 20, and 40 mg/kg soybean isoflavones (SI) for 42 days. At 21 days of age, chickens were inoculated with an infectious bursal dose (causing 50% morbidity) of the infectious bursal disease virus (IBDV) BC 6/85 strain by the eye-drop and nasal route (except for the non-infected group). Average daily gain (ADG) and average daily feed intake (ADFI) decreased (*p* < 0.05) in broilers infected with infectious bursal disease virus (IBDV) from 22 to 42 days. However, infected broilers fed 10 and 20 mg SI/kg had the maximum (*p* <0.05) ADG and ADFI from 1 to 42 days. Body weight (BW) increased (*p* < 0.05) in infected broilers fed the 10 and 20 mg SI /kg diet. The bursa weight at 7 days post-infection (dpi) was increased (*p* < 0.05) by the supplemental 10 mg SI/kg diet. Infected broilers showed the highest (*p* < 0.05) bursa lesions, with an average score of 4.0 ± 0.0, while the severity of bursa lesions was decreased (*p* < 0.05) at 3 dpi and 7 dpi by the supplemental 20 mg SI/kg diet. Supplemental SI at 20 mg/kg decreased (*p* < 0.05) the viral protein 5 (VP5) mRNA expression at 3 dpi and 7 dpi. The level of interferon gamma (IFNγ) was elevated (*p* < 0.05) in the infected group at 3 dpi and 7 dpi as compared with the control group, while its level was decreased *(p* < 0.05) by supplemental 10 mg/kg SI at 3 dpi. The level of nuclear factor κB in the bursal tissue showed the lowest value (*p* < 0.05) with supplemental 10 and 20 mg SI/kg diet at 7 dpi. Supplemental 10, 20, 40 mg/kg SI improved (*p* < 0.05) the serum total antioxidant activity (T-AOC) in infected broilers at 3 dpi. In addition, the serum level of malondialdehyde (MDA) decreased (*p* < 0.05) in the group fed 20 mg/kg SI at 7 dpi. In conclusion, supplemental 10~20 mg/kg SI may have a positive effect on broiler chickens infected with IBDV, probably because SI decrease the severity of bursa lesions and viral protein 5 mRNA expression, and have strong antioxidant activity.

## 1. Introduction

Infectious bursal disease (IBD), or Gumboro disease, is an acute, highly contagious disease of young chickens caused by the infectious bursal disease virus (IBDV), characterized by inflammation and subsequent atrophy of the bursa of Fabricius, and immune suppression and mortality, generally at 3 to 6 weeks of age [1,2,3,4]. It has been shown that IBDV induces suboptimal feed conversion and weight gain [5]. In addition, immune dysfunction decreases the growth performance and increases carcass condemnation rates, but increases the rate of mortality and morbidity due to secondary viral and bacterial infections [6].

Recently, bioactive compounds in feedstuffs or feed additives are considered as an important strategy to boost immunity in modern poultry production [7,8,9,10]. Isoflavones are natural molecules available in edible plants, particularly in soybeans, red clover, and kudzu root [11,12]. Isoflavones, as phenolic compounds, are the main phytoestrogens of soybeans [13]. Isoflavones, including genistein, daidzein, and glycitein, are similar in structure to 17-β-estradiol. Soy isoflavones are used as a supplement to improve growth performance, antioxidant activity, and immune function [14,15,16,17,18,19]. These reasons caused us to hypothesize that supplemental soy isoflavones may improve the performance and immune function of broilers chickens infected with IBDV.

This study was conducted to investigate the ability of isoflavones in the amelioration of oxidative stress and immune function of broilers chickens challenged with IBDV.

## 2. Materials and Methods

The experimental protocol was reviewed and approved by the Institute of Animal Science, Guangdong Academy of Agricultural Sciences, China (GAASISA-2015-03).

### 2.1. Birds, Virus, and Diets

A total of 200 one-day-old Lingnan yellow-feathered male broilers were obtained from a commercial hatchery Guangdong Wiz Agricultural Science and Technology Co., Guangzhou, China) and raised under standard conditions with free access to water and feed. The strain of IBDV, BC 6/85, is a classic strain of virulent IBDV used as a standard challenge strain in China and was purchased from the China Institute of Veterinary Drug Control (Haidian District, Beijing, China). Nutrient levels of the diets were based on the National Research Council [20] recommended nutrient requirements for broiler chickens (Table 1)

### 2.2. Experimental Design

On the first day of the experiment, 200 one-day-old yellow-feathered male broiler chickens were weighed and allotted randomly to five treatment groups, each of which included four replicates of 10 birds. Broilers were placed in floor pens (1 × 2 m). The litter thickness was 5 cm of sawdust. All birds were fed the same basal diet, supplemented with 0 (non-infected control group), 0 (infected control), 10, 20, or 40 mg/kg soybean isoflavones (SI) (supplied by Newland Feed Science and Technology Co., Guangdong, China).These treatments are described as non-infected control, IBDV (0 SI), IBDV (10 SI), IBDV (20 SI), and IBDV (40 SI), respectively (Table 2). 

At 21 days of age, chickens were inoculated with the bursal infectious dose causing 50% morbidity of the IBDV BC 6/85 strain by the eye-drop and nasal route, except for the non-infected control group. 

A pre-experiment had been conducted to titrate the optimal dose of the inoculation. By administering the chosen dose, visible pathological changes were visible on the bursa of Fabricius at 5 days post-infection (dpi) without evident mortality. During the experiment, which lasted 42 days, the infected and non-infected groups of chickens were housed in equivalent but separate places. 

### 2.3. Growth Performance

Broiler chickens were weighed at 1, 21, and 42 days of age. Average daily feed intake was determined on a per pen basis. The feed conversion ratio (FCR) was calculated. Mortality and health status were recorded daily.

### 2.4. Blood Sampling and Laboratory Analyses 

Eight broilers per treatment group (two birds per replicate) were selected randomly and bled into tubes (5 mL per bird) from a wing vein at 3 and 7 dpi to collect the serum, and then the broilers were slaughtered and bursa of Fabricius was collected and weighed from each broiler. The half of bursa was fixed in 4% buffered formaldehyde. Another half was snap-frozen with liquid nitrogen and stored at −80°C to analyze viral protein 5 (VP5) mRNA expression.

### 2.5. Histology of Bursa of Fabricius

The collected tissues of bursa of Fabricius were fixed with 4% formaldehyde solution for 24 h. Serial sections were cut at 5 μm were dehydrated, cleared, embedded in paraffin, deparaffinized in xylene, rehydrated, and stained with hematoxylin and eosin. Three sections were made for each sample to observe the bursal lesion and to measure the degree of damage of the bursal follicle using light microscope. To observe the bursal lesion and to measure the degree of damage of the bursal follicle, the histopathological changes of the bursa of Fabricius were scored according to the methods of Sharma et al. [21] and Kim et al. [22]: 0 = normal bursa of Fabricius; 1 = 1–25%; 2 = 26–50%; 3 = 51–75%; and 4 = 76–100% of follicles showing cellular depletion. 

### 2.6. Viral Protein 5 (VP5) mRNA Expression

Total RNAs was extracted from the Bursal homogenates using QiAmp Viral RNA Mini kit (Qiagen GmbH, Hilden, Germany) according to the manufacturer’s instructions. Gene-specific primers for viral protein 5 (VP5) and the endogenous reference gene (β-actin) are shown in Table 3. Briefly, the 50-µL reaction mixture contained 10 µL of extracted RNA, 10 µL of 5· RT-PCR buffer, 2 µL primer F, 2 µL primer R, 2 µL dNTP mix containing 400 µM each of dATP, dGTP, dCTP, and dTTP, and 2 µL of Qiagen One Step Enzyme Mix. A fragment of 94 bp of the 5′ noncoding region was amplified using a PCR reaction with the SYBR Premix PCR kit (Takara, Dalian, China). The PCR program was 95°C for 10 min followed by 40 cycles of 95°C for 15 s and 60 °C for 60 s. The standard curve was generated using pooled samples and efficiency was calculated from standard curves. Each sample was run in duplicate and a no-template control was included. Specificity of the amplification was verified via melting curve analysis and the specificity of the product was confirmed by electrophoresis on a 1.2% agarose gel, with purification using a DNA purification kit (Takara), and sequencing (Shanghai Sangon Biotech Co. Ltd., Shanghai, China). The difference of the cycle threshold (Ct) value for the 18s rRNA was less than 0.5 across all treatments, and therefore was considered to be an appropriate endogenous control. Average gene expression relative to the endogenous control for each sample was calculated using the 2^−ΔΔ*C*t^ method [23]. The calibrator for each studied gene was the average ^Δ^*C*t value of the control group.

### 2.7. Determination of Antioxidant Capacity in the Serum

Serum total antioxidant activity (T-AOC) and malonaldehyde (MDA) were measured spectrophotometrically. A total antioxidant capacity assay kit (A015–1, Nanjing Jiancheng Bioengineering Institute, Nanjing, China) was used according manufacturer’s instructions and was expressed in U/mL. The MDA levels were assayed using the thiobarbituric acid method [24], reading the absorbance at 532 nm with the spectrometer. 

### 2.8. Determination of Bursal Immunologic Indices

Bursa of Fabricius samples were thawed at 4°C and homogenized in 10 volumes of cold normal saline. The homogenates were then centrifuged at 20,000× *g* for 20 min at 4 °C and the supernatant was collected for analyses. Diluent solution and standard samples were added at 100 μL per well in duplicate wells. The plate was incubated for 2 h at 37 °C, followed by three washings with wash solution. The immunological indicators of interleukin-2 (IL-2), interleukin-6 (IL-6), interferon gamma) IFNγ), and nuclear factor κB were (NF-κB) determined by ELISA kits. The kits were purchased from Shanghai Jianglai Biotechnology Co., Ltd. (Shanghai, China), and the specific operation was carried out according to the instructions. 

### 2.9. Statistical Analyses

The replicate was the experimental unit. The effects of SI supplementation levels were examined by one-way ANOVA using the general linear model GLM procedures of SAS software (v9.2, SAS Institute, Cary, NC, USA). In the absence of SI, IBDV-infected and non-infected controls were compared by *t*-tests. Significance was declared at *p* < 0.05. All data are expressed as means ± SE.

## 3. Results and Discussions

Some of the main strategies during stress periods such as viral infections are to boost the immune function, maximize antioxidant ability, and minimize lipid peroxidation. Therefore, this study was conducted to investigate the ability of isoflavones in the amelioration of oxidative stress and in the immune function of broilers chickens challenged with IBDV. 

### 3.1. Bursa of Fabricius

The effect of SI supplementation on bursa of Fabricius weight and index of broilers challenged with infectious bursal disease virus is shown in Table 4. In addition, the bursa lesion score is shown in Table 5.

The weight (g) and the index of bursa of Fabricius (%) were reduced significantly *(p* < 0.05) in broiler chicks infected with IBDV as compared with those of the control group (non-infected) at 3 dpi and 7 dpi. However, supplemental 10 mg of SI increased the bursa weight significantly *(p* < 0.05) at 7 dpi. Our finding is in agreement with the findings of Li et al. [25]. They reported that a significant decrease in the bursa to body weight ratios (B/BW) had appeared at 7 dpi. Bursa lesions in infected broiler had an average score of 4.0 ± 0.0 compared with non-infected control (*p* < 0.05). However, the severity of bursa lesions was decreased (*p* < 0.05) at 3 dpi and 7 dpi by the supplemental 20 mg SI/kg diet. This is in agreement with the result of Li et al. [25]. They found that bursa lesion score was 4.0 ± 0.0 at 3 dpi in IBDV-infected group. 

**Figure 1:**
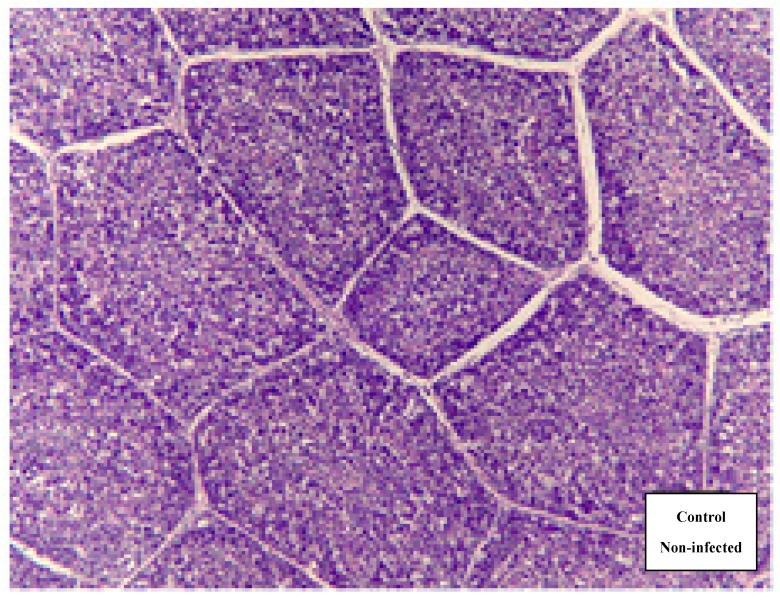
Normal bursal tissue section stained with hematoxylin and eosin (H&E) in the non-infected group.

**Figure 2:**
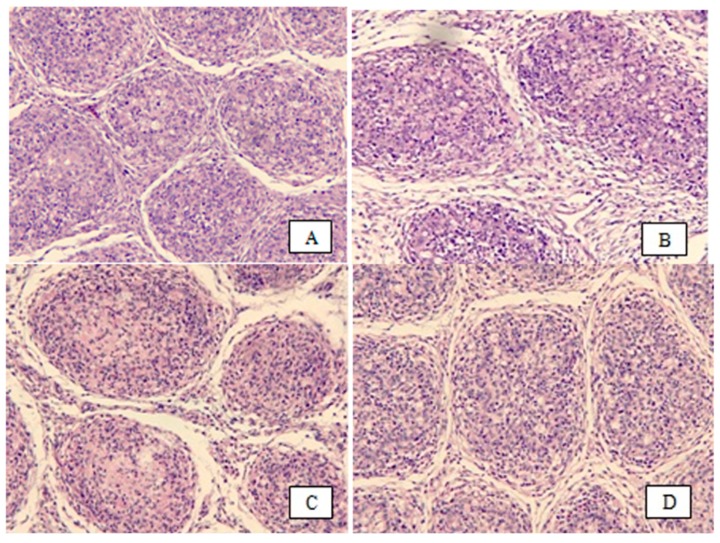
Bursal tissue section stained with hematoxylin and eosin (H&E) of each group at 3 dpi (200×). (**A**) is from the IBDV (0 SI); (**B**) is from the IBDV (10 SI); (**C**) is from the IBDV (20 SI); and (**D**) is from the IBDV (40 SI).

**Figure 3:**
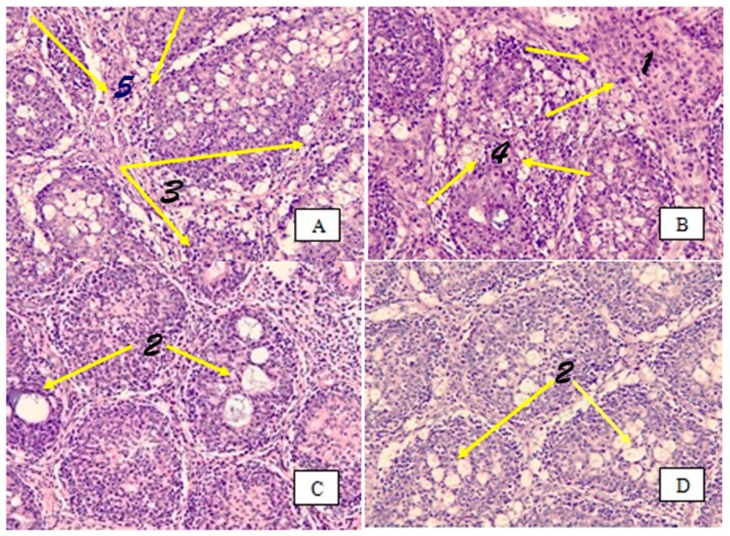
Bursal tissue section stained with hematoxylin and eosin (H&E) of each infected group at 7 dpi (200×). (**A**) is from the IBDV (0 SI); (**B**) is from the IBDV (10 SI); (**C**) is from the IBDV (20 SI); and (**D**) is from the IBDV (40 SI). (1) Bursa microscopically revealed complete loss of architecture. (2) Bursa with infectious bursitis presenting necrosis and heterophil invasion. (3) The lining epithelium was highly corrugated. (4) There was no intact lymphoid follicle. (5) The entire area was filled up by fibrous tissue. The IBDV+20 SI group was the best among the infected groups (the architecture is almost clear between follicles, the lining epithelium was less corrugated, and there was less necrosis, heterophil invasion, and fibrous tissue).

In terms of bursal damage, the control group (non-infected) had no signs of bursal damage (Figure 1), while all infected broilers had bursal damage at 3 dpi and 7 dpi (Figure 2 and Figure 3). This finding is in agreement with that of Li et al. [25], who demonstrated a depletion of lymphoid cells in bursal follicles was observed microscopically. In the present study, infected broilers fed a 20 mg SI/kg diet had the lowest amount of bursal damage. As shown in Figure 3, the architecture is almost clear between the follicles, the lining epithelium was less corrugated, there was less necrosis and heterophil invasion, and fewer fibrous tissues were observed. It has been shown that IBDV infection induced a temporary or permanent destruction of the bursa of Fabricius and other lymphoid organs [26,27]. Destruction of B cells contributes to IBDV-induced immune suppression [28].

### 3.2. Viral Protein 5 (VP5) mRNA Expression

Viral protein 5 (VP5) expression was higher (*p* < 0.05) in broilers infected with IBDV as compared with those of the control group at 3 dpi and 7 dpi (Table 6). However, supplemental 20 mg/kg of SI reduced VP5expression at 3 dpi and 7 dpi. It has been indicated that RT-PCR was a sensitive test to detect the IBDV [2,29,30]. According to our knowledge, no previous study investigated the effects of SI on VP5. VP5 is one protein employed by IBDV to induce the programmed cell death process [31]. It has been suggested that VP5 might play an important role as anti-apoptotic protein at an early stage of IBDV infection [32]. In addition, Qin and Zheng [33] suggested that VP5 as an anti-apoptotic protein is an important factor to support viral replication at the early stage of IBDV infection. The anti-apoptotic activity of VP5 was noticed at 8 or 12 h post-infection [34], while VP5 induced apoptosis were found after 24 h post-infection [35].

### 3.3. Immune Function

Effect of adding soy isoflavones on immunity of IBDV-challenged broilers is presented in Table 7.

The bursal concentration of interleukin 2 (IL-2) was lower *(p* < 0.05) in challenged broilers, while supplemental 20 mg of SI increased its level *(p* < 0.05) at 3 dpi, which is considered an acute stage of IBDV infection. IL-2 is a cytokine secreted by activated T lymphocytes, which has an important role in regulation of host response to pathogenic challenge [36]. In addition, the bursal concentration of interleukin 6(IL-6) was lower (*p* < 0.05) in the infected group, while supplemental 10 mg of SI increased its level (*p* < 0.05) at 3 dpi.

Long et al. [37] reported that IBDV infection increased IFN-γ mRNA relative expression in the bursa of Fabricius. Interferon-γ is one of the proinflammatory Th1 cytokines [38]. The level of interferon gamma (IFNγ) in the bursa of Fabricius was elevated (*p* < 0.05) in infected broilers with IBDV at 3 dpi and 7 dpi as compared with the control group, while its level was decreased (*p* < 0.05) by supplemental 10 mg/kg SI at 3 dpi. In the current study, the effect of dietary SI in immune function was significant during early stages of infection, and it was obviously significant at 10 and 20 mg SI/kg diet. It is well known that infectious bursal disease (IBD) disease peaks between 2 to 5 day post infection and is practically cleared by day 7 [39].

The bursal level of nuclear factorκB (NF-κB) was higher in infected broilers with IBDV at 7 dpi, while its level was decreased (*p* < 0.05) by supplemental 10 and 20 mg SI at 3 dpi and 7 dpi (Table 8). It has been shown that SI are associated with cell survival, cell cycle, inflammation, and apoptosis, and they suppress nuclear factor (NF)-κB and other signaling pathways [40]. In addition, it has been reported that NF-κB activity was blunted more efficiently by genistein, probably due to its additional antioxidant effect [41]. In the present study, supplemental 10, 20, and 40 mg/kg SI significantly (*p* < 0.05) improved the serum T-AOC in infected broilers at 3 dpi (Table 8). In addition, the serum level of MDA was decreased (*p* < 0.05) in the group fed 20 mg/kg SI at 7 dpi. 

Huang et al. [19] reported that SI improved the immune function in young piglets fed oxidized fish oil. Moreover, Lv et al. [42] reported that genistein (GEN boosted the anti-viral capacity of broilers chickens. They reported that the Newcastle disease (ND) and IBD antibody titers in the GEN group were higher (*p* < 0.05) than broilers in the control group.

### 3.4. Antioxidant Capacity and Oxidative Stress

In the present experiment, supplemental 10, 20, and 40 mg/kg SI significantly (*p* < 0.05) improved the serum T-AOC in infected broilers at 3 dpi (Table 8). It has been reported that SI have antioxidant properties via detoxifying free radical species and up-regulating antioxidant genes [40].The serum level of MDA was decreased(*p* < 0.05) in the group fed 20 mg/kg SI at 7 dpi. The level of MDA can be a marker of the level of lipid peroxidation endogenously, which is the result of diminished antioxidant protection as levels of reactive oxygen species and antioxidants ROS increase or there is weak antioxidant activity.

### 3.5. Growth Performance

Infected broilers with IBDV had decreased (*p* < 0.05) average daily gain (ADG) and average daily feed intake (ADFI) from 22 to 42 days. However, infected broilers fed 10 and 20 mg/kg had the maximum *(p* < 0.05) ADG and ADFI from 1 to 42 days. In addition, body weight (BW) was increased (*p* < 0.05) in infected broilers fed 10 and 20 mg/kg (Table 9).

It has been reported that IBDV decreased weight gain and feed efficiency [5]. Recently, Wang and Wu [43] reported that SI alleviated the growth suppression induced by dextran sulfate sodium in mice. In addition, Greiner et al. [44,45] reported that soybean genistein (200 mg/kg) and daidzein (200 or 400 mg/kg) could improve growth in virally challenged pigs. There is a positive effect of SI on infected broiler chickens with IBDV, probably because SI decrease the severity of bursa lesions and viral protein 5 mRNA expression, and have strong antioxidant activity.

In the present study, no broilers died due to IBDV infection. These findings are in agreement with other studies [46,47].

## 4. Conclusions

Supplemental 10~20 mg/kg SI may have a positive effect on broiler chickens infected with IBDV, probably because SI decrease the severity of bursa lesions andviral protein 5 mRNA expression, and have strong antioxidant activity.

## Figures and Tables

**Table 1:** Ingredient and composition of the basal diets for Chinese yellow-feathered broilers at 1–21 and 22–42 days of age (as fed-basis).

Ingredients	Composition, g/kg	
	1–21 Days of Age	22–42 Days of Age
Maize	584.0	608.0
Wheat bran	43.0	38.0
Fish meal	22.0	10.0
Soybean meal	264.0	220.0
Maize gluten meal	20.0	30.0
Soybean oil	13.0	29.0
Lysine	0.0	1.0
Methionine	1.0	0.8
Limestone	12.7	12.0
Dicalcium phosphate	15.1	14.5
Salt	2.5	2.5
Zeolite	12.7	24.2
Vitamin-mineral premix ^1^	10.0	10.0
Total	1000.0	1000.0
**Chemical composition, g/kg ^2^**		
Crude protein	199.3	187.4
Lysine	10.5	9.8
Methionine	4.6	4.0
Calcium	10.0	9.0
Non-phytate P	4.5	4.0
Metabolizable Energy, MJ/kg	12.13	12.55

^1^ Supplied per kilogram of diet: vitamin A, 14,700 IU; vitamin D_3_, 3300 IU; vitamin E, 20 IU; vitamin K_3_, 3.9 mg; vitamin B_1_, 3 mg; vitamin B_2_, 9.6 mg; vitamin B_6_, 6 mg; vitamin B_12_, 0.03 mg; nicotinic acid, 60 mg; pantothenic acid, 18 mg; folic acid, 1.5 mg; biotin, 0.36 mg; FeSO_4_⋅7H_2_O, 80 mg; CuSO_4_⋅5H_2_O, 8 mg; MnO, 80 mg; KI, 0.38 mg; and NaSeO_3_, 0.44 mg. The carrier was zeolite. ^2^ Values were calculated from data provided by Feed Database in China (2016) except that crude protein was analysed.

**Table 2:** The design of the experimental study.

Treatments	Control	IBDV (0 SI)	IBDV (10 SI)	IBDV (20 SI)	IBDV (40 SI)
SI, mg/kg	0	0	10	20	40
IBDV	−	+	+	+	+

SI: synthetic soybean isoflavones; IBDV: infectious bursal disease virus.

**Table 3:** Sequences of primers for the real-time PCR.

Gene name	Primer Sequence	Amplicon Size [bp]
**VP5 ^1^**	F:5′-GAGCCTTCTGATGCCAACAAC-3′ R:5′-CAAATTGTAGGTCGAGGTCTCTGA-3′	94 bp
**β-actin**	F:5′-TGGCATTGCTGACAGGAT-3′ R:5′-CTGCTTGCTGATCCACAT-3′	160 bp

^1^ viral protein 5(encodes a 17-kDa non structural polypeptide).

**Table 4:** Effect of adding soy isoflavones on bursa development of IBDV-challenged broilers ^1^.

Indices		Treatments				
		Control	IBDV (0 SI)	IBDV (10 SI)	IBDV (20 SI)	IBDV (40 SI)
Bursa weight, g	3days PI	2.45 ± 0.15 ^A^	1.60 ± 0.05 ^B^	1.88 ± 0.18	1.88 ± 0.17	1.80 ± 0.10
	7days PI	2.25 ± 0.22 ^A^	0.58 ± 0.06 ^Bb^	0.87 ± 0.05 ^a^	0.63 ± 0.08 ^b^	0.59 ± 0.05 ^b^
Bursa index, %	3days PI	0.46 ± 0.03 ^A^	0.32±0.02 ^B^	0.34 ± 0.04	0.36 ± 0.04	0.35 ± 0.02
	7days PI	0.32 ± 0.02 ^A^	0.10±0.01 ^B^	0.14 ± 0.00	0.13 ± 0.02	0.10 ± 0.01

^1^ Data are means of eight broilers chickens per treatment (two broilers/replicate). Capital letters indicate statistically significant (*p* < 0.05) differences between control group and IBDV group by Student’s *t*-test; small letters indicate statistically significant (*p* < 0.05) differences between IBDV (0 SI), IBDV (10 SI), IBDV (20 SI) and IBDV (40 SI).

**Table 5:** Effect of adding soy isoflavones on bursa lesion score of IBDV-challenged broilers ^1^.

Indices		Treatments				
		Control	IBDV (0 SI)	IBDV (10 SI)	IBDV (20 SI)	IBDV (40 SI)
Bursa Score	3days PI	0.00±0.00 ^B^	4.00 ± 0.00 ^Aa^	3.63 ± 0.26 ^ab^	3.13 ± 0.30 ^b^	3.75 ± 0.16 ^ab^
	7days PI	0.00±0.00 ^B^	3.88 ± 0.13 ^Aa^	4.00 ± 0.00^a^	3.13 ± 0.30 ^b^	3.86 ± 0.14 ^a^

^1^ Data are means of eight broilers chickens per treatment (two broilers/replicate). Capital letters indicate statistically significant (*p* < 0.05) differences between the control group and IBDV group by Student’s *t*-test; small letters indicate statistically significant (*p* < 0.05) differences between IBDV (0 SI), IBDV (10 SI), IBDV (20 SI) and IBDV (40 SI).Note: Histopathological score of bursa of Fabricius: 0 = normal bursa of Fabricius; 1 = 1–25%; 2 = 26–50%;3 = 51–75%; and 4 = 76–100% of follicles showing cellular depletion.

**Table 6:** Effect of adding soy isoflavones on the IBDV mRNA expression of bursal tissue in IBDV—challenged broilers ^1^.

Indices		Treatments				
		Control	IBDV (0 SI)	IBDV (10 SI)	IBDV (20 SI)	IBDV (40 SI)
viral protein 5 [VP5]	3days PI	0.00 ± 0.00 ^B^	0.0554 ± 0.0211 ^Aa^	0.0492 ± 0.0080 ^a^	0.0052 ± 0.0026 ^b^	0.0548 ± 0.0197 ^a^
	7days PI	0.00 ± 0.00 ^B^	0.0025 ± 0.0005 ^Aa^	0.0012 ± 0.0003 ^b^	0.0010 ± 0.0004 ^b^	0.0034 ± 0.0004 ^a^

^1^ Data are means of eight broilers chickens per treatment (two broilers/replicate). Capital letters indicate statistically significant (*p* < 0.05) differences between control group and IBDV group by Student’s *t*-test; small letters indicate statistically significant (*p* < 0.05) differences between IBDV (0 SI), IBDV (10 SI), IBDV (20 SI), and IBDV (40 SI).

**Table 7:** Effect of adding soy isoflavones on bursal immune response of IBDV-challenged broilers ^1^.

Indices		Treatments				
		Control	IBDV (0 SI)	IBDV (10 SI)	IBDV (20 SI)	IBDV (40 SI)
IL-2, pg/mL	3d PI	106.52 ± 5.55	99.90 ± 3.34 ^b^	104.05 ± 3.91 ^ab^	111.90 ± 2.99 ^a^	113.32 ± 3.43 ^a^
	7d PI	216.07 ± 14.27	201.13 ± 9.86 ^b^	220.39 ± 15.57 ^b^	195.21 ± 9.47^b^	270.04 ± 24.25 ^a^
IL-6, pg/mL	3d PI	50.22 ± 5.62	40.24 ± 3.14 ^b^	53.61 ± 4.78 ^a^	48.29 ± 2.78 ^ab^	47.41 ± 2.53 ^ab^
	7d PI	33.71 ± 4.47 ^B^	56.78 ± 6.02 ^A^	47.58 ± 5.09	51.43 ± 2.87	55.31 ± 3.24
IFNγ, ng/mL	3d PI	7.95 ± 1.05 ^B^	12.94 ± 0.85 ^Aa^	9.69 ± 0.52 ^b^	10.34 ± 1.34 ^ab^	9.24 ± 1.06 ^b^
	7d PI	5.58 ± 0.24 ^B^	7.64 ± 0.96 ^A^	8.53 ± 0.38	6.49 ± 0.89	6.38 ± 1.14
NF-κB, pg/mL	3d PI	54.26 ± 8.67	50.64 ± 7.29	58.39 ± 9.80	67.52 ± 7.29	50.50 ± 6.90
	7d PI	58.91 ± 1.11 ^B^	113.08 ± 9.50 ^Aa^	68.93 ± 6.42 ^b^	80.56 ± 13.31 ^b^	92.85 ± 8.93 ^ab^

^1^ Data are the means of eight broilers chickens per treatment (two broilers/replicate). IL-2; interleukin 2; IL-6: interleukin 6; IFNγ: interferon gamma; NF-κB: nuclear factorκB. Capital letters indicate statistically significant (*p* < 0.05) differences between the control group and IBDV group by Student’s *t*-test; small letters indicate statistically significant (*p* < 0.05) differences between IBDV (0 SI), IBDV (10 SI), IBDV (20 SI) and IBDV (40 SI).

**Table 8:** Effect of adding soy isoflavones on serum antioxidant index of IBDV-challenged broilers ^1^.

Indices ^2^		Treatments				
		Control	IBDV (0 SI)	IBDV (10 SI)	IBDV (20 SI)	IBDV (40 SI)
TAOC, U/mL	3days PI	9.86 ± 0.36 ^A^	4.20 ± 0.20 ^Bb^	7.30 ± 0.33 ^a^	6.63 ± 0.54 ^a^	6.84 ± 0.45 ^a^
	7days PI	7.70 ± 0.63	7.60 ± 0.61	7.24 ± 0.49	7.30 ± 0.43	6.66 ± 0.52
MDA, mol/mL	3days PI	5.19 ± 0.57	4.12 ± 0.55	5.21 ± 0.81	4.40 ± 0.98	3.18 ± 0.68
	7days PI	4.33 ± 0.21	4.45 ± 0.75 ^a^	3.37 ± 0.57 ^ab^	2.45 ± 0.30 ^b^	2.07 ± 0.26 ^b^

^1^ Data are means of eight broilers chickens per treatment (two broilers/replicate). ^2^ Total antioxidant activity (T-AOC), malonaldehyde (MDA). Capital letters indicate statistically significant (*p* < 0.05) differences between control group and IBDV group by Student’s *t*-test; small letters indicate statistically significant (*p* < 0.05) differences between IBDV (0 SI), IBDV (10 SI), IBDV (20 SI), and IBDV (40 SI).

**Table 9:** Effect of adding soy isoflavones on growth performance of IBDV-challenged broilers ^1^.

Indices		Treatments				
		Control	IBDV (0 SI)	IBDV (10 SI)	IBDV (20 SI)	IBDV (40 SI)
BW, g	1	39.55 ± 0.19	39.75 ± 0.17	40.00 ± 0.07	39.93 ± 0.20	39.70 ± 0.14
	21	455.00 ± 2.04	452.50 ± 14.22 ^ab^	473.75 ± 5.54 ^a^	460.00 ± 2.89 ^ab^	436.25 ± 9.87 ^b^
	42	1351.50 ± 16.05	1288.04 ± 27.34 ^ab^	1320.58 ± 13.98 ^a^	1332.06 ± 23.64 ^a^	1228.58 ± 40.72 ^b^
Average daily gain, g	1–21	20.77 ± 0.10	20.64 ± 0.71 ^ab^	21.69 ± 0.28 ^a^	21.09 ± 0.11 ^ab^	19.83 ± 0.49 ^b^
	22–42	40.75 ± 0.79 ^A^	37.98 ± 0.73 ^B^	38.49 ± 0.66	39.57 ± 1.04	36.02 ± 1.69
	1–42	31.23 ± 0.39	29.72 ± 0.65 ^ab^	30.49 ± 0.33 ^a^	30.76 ± 0.56 ^a^	28.31 ± 0.97 ^b^
ADFI, g	1–21	34.05 ± 0.32	34.26 ± 0.37 ^b^	35.87 ± 0.43 ^a^	34.78 ± 0.22 ^b^	32.38 ± 0.24 ^c^
	22–42	77.55 ± 1.57 ^A^	73.23 ± 0.42 ^Bb^	77.11 ± 1.21 ^a^	77.90 ± 1.43 ^a^	71.79 ± 1.11 ^b^
	1–42	56.84 ± 0.74	54.67 ± 0.80 ^ab^	57.47 ± 0.56 ^a^	57.37 ± 0.66 ^a^	52.18 ± 1.28 ^b^
FCR, g:g	1–21	1.63 ± 0.00	1.67 ± 0.06	1.66 ± 0.01	1.66 ± 0.01	1.64 ± 0.03
	22–42	1.92 ± 0.00	1.93 ± 0.04	2.01 ± 0.03	1.95 ± 0.02	2.00 ± 0.06
	1–42	1.83 ± 0.00	1.84 ± 0.01	1.89 ± 0.02	1.85 ± 0.02	1.85 ± 0.02

^1^ Data are means of four replications per treatments, with 10 broilers per replicate. Capital letters indicate statistically significant (*p* < 0.05) differences between the control group and IBDV group by Student’s *t*-test; small letters indicate statistically significant (*p* < 0.05) differences between IBDV (0 SI), IBDV (10 SI), IBDV (20 SI), and IBDV (40 SI). ADFI: average daily feed intake; FCR: feed conversion ratio.
